# Neck Circumference Positively Relates to Cardiovascular Risk Factors in College Students

**DOI:** 10.3390/ijerph15071480

**Published:** 2018-07-13

**Authors:** Oluremi A. Famodu, Makenzie L. Barr, Sarah E. Colby, Wenjun Zhou, Ida Holásková, Miriam P. Leary, Carol Byrd-Bredbenner, Anne E. Mathews, Melissa D. Olfert

**Affiliations:** 1 Division of Animal and Nutritional Sciences, West Virginia University, 1194 Evansdale Dr., Agricultural Sciences Building, Morgantown, WV 26506, USA; OluremiFamodu@tcomn.com (O.A.F.); mbarr6@mix.wvu.edu (M.L.B.); miriam.leary@mail.wvu.edu (M.P.L.); 2 Department of Nutrition, The University of Tennessee, 1215 W. Cumberland Avenue, 229 Jessie Harris Building, Knoxville, TN 37996-1920, USA; scolby1@utk.edu; 3 Department of Business Analytics and Statistics, The University of Tennessee, 916 Volunteer Blvd., Knoxville, TN 37996-1920, USA; wzhou7@gmail.com; 4 Office of Statistics, West Virginia University, Davis College of Agriculture, Natural Resources and Design, West Virginia Agriculture and Forestry Experiment Station, 1194 Evansdale Dr., Agricultural Sciences Building, Morgantown, WV 26506, USA; ida.holaskova@mail.wvu.edu; 5 Department of Nutritional Sciences, Rutgers University, 26 Nichol Ave., New Brunswick, NJ 08901, USA; bredbenner@sebs.rutgers.edu; 6 Food Science and Human Nutrition Department, University of Florida, 572 Newell Dr., 359 FSHN Building, P.O. Box 110370, Gainesville, FL 32611-0370, USA; Anne.mathews@ufl.edu

**Keywords:** neck circumference, young adult, risk factor, obesity

## Abstract

The objective of this study was to determine the relationship between neck circumference (NC) and other anthropometric measures and examine cut-off points for males and females according to existing waist circumference cut-off levels in this age group. Across 8 universities, 1562 students underwent a physical assessment. Spearman rho correlations (ρ) were calculated to determine associations between NC and other continuous variables of health. Receiving operating characteristic curves were constructed to assess the optimal cut-off levels of NC of males and females with central obesity. Participants were predominantly Caucasian (67%), female (70%), and outside of Appalachia (82%). Forty-one percent of males and 34% of females had a BMI ≥ 25 kg/m^2^. In both sexes, significant positive correlations were seen between NC and body weight, BMI, waist circumference, hip circumference, and systolic blood pressure (all *p*-values < 0.0001). NC ≥ 38 cm for males and ≥33.5 cm for females were the optimal cut-off values to determine subjects with central obesity. NC has been identified to closely correlate with other anthropometric measurements related to disease and could be used as a convenient, low-cost, and noninvasive measurement in large-scale studies.

## 1. Introduction

The massive healthcare and economic burdens associated with the obesity epidemic is projected to worsen with another 65 million adults becoming obese by 2030. This equates to an additional $48–66 billion/year in United States (U.S.) healthcare costs [1]. The health burden associated with obesity is primarily driven by an increased risk of cardiovascular disease, type 2 diabetes, and some cancers [2,3]. Clearly, promoting healthier weights is necessary for disease prevention and reducing associated economic burdens.

A primary component of disease prevention is risk factor identification in those who appear to be asymptomatic. Young adults often appear healthy despite having multiple risk factors for obesity-related diseases. Therefore, risk factor screening during young adulthood is important for disease prevention, and also offers the opportunity to establish a lifetime of health-promoting habits [4,5]. A traditional screening tool for health-related risk factors is body mass index (BMI, weight (k)/height (m) squared), which is a quick, easy, and inexpensive tool used to classify individuals as underweight (BMI < 18.5 kg/m^2^), normal weight (18.5 kg/m^2^ ≤ BMI < 24.9 kg/m^2^), overweight (25 kg/m^2^ ≤ BMI < 29.9 kg/m^2^), or obese (BMI ≥ 30 kg/m^2^) [6]. While BMI is widely used and accepted as a measure of overweight/obesity in the U.S., its indirect measurement of body fat can result in misclassification [7]. Despite the relationship between upper-body subcutaneous adipose tissue distribution, or central adiposity, and increased risk of cardiovascular disease and metabolic dysfunction, BMI does not differentiate between body fat distribution patterns [8,9,10].

Gold standard measures of obesity and body fat distribution include ultrasound, computed tomography, and magnetic resonance imaging. However, these methods are expensive and often impractical in large-scale studies; therefore, reliable, simple, and easily implemented methods of obesity and body fat distribution are needed. Neck circumference (NC) has been used as a biomarker of central adiposity, a body fat distribution pattern associated with metabolic dysfunction, including glucose intolerance, hyperinsulinemia, diabetes, and hypertriglyceridemia [11,12]. Evidence indicates the NC is a strong indicator of elevated serum triglycerides and decreased serum high-density lipoprotein (HDL) cholesterol compared to BMI and waist circumferences (WC) in both sexes [13]. Additionally, NC has been used to evaluate relationships with cardiovascular risk, insulin resistance, and biochemical components of metabolic syndrome [14,15]. As a result, this often overlooked method of evaluating body fat can be useful in screening health risk for young adults, especially for large samples when noninvasive, inexpensive, and easily implemented measures are needed.

Previous studies have found NC cut-offs to determine central obesity, but not in a population of young, U.S. adults [16,17]. Therefore, this study aimed to compare NC to other anthropometrics in young, U.S. adults and determine NC cut-off points for males and females according to pre-existing, age-specific WC cut-off levels. WC was chosen in the current study for the reference point due to its close association with central obesity and risk of other comorbidities [18].

## 2. Materials and Methods

### 2.1. Test Sample

This cross-sectional study was conducted at eight universities (West Virginia University, Auburn University, South Dakota State University, University of Maine, Florida University, Kansas State University, Syracuse University, and University of Tennessee) across the United States under a 5-year National Institute of Food and Agriculture, U.S. Department of Agriculture funded research project (#2014-67001-21851) called *Get Fruved* (NCT 02941497 on clinicaltrials.gov). Data was collected on a convenience sample of college students (*n* = 1562) aged 18–28 years old during the fall of 2014 and spring of 2015. Each university’s IRB approved the study procedures and all participants provided written consent to participate by signing an IRB-approved informed consent form prior to participating. This study was retrospectively registered on 21 October 2016 on clinicaltrials.gov, NCT02941497.

### 2.2. Anthropometry

Measurements were taken in duplicate by the same researcher and results were averaged for analysis. NC was measured immediately below the laryngeal prominence (the Adam’s Apple), using a Gulick tape measure (North Coast Medical; Gilroy, CA, USA), while standing erect with eyes facing forward [19]. Standing height and body weight were measured using a wall-mounted Stadiometer (SECA 213) and digital floor scale (Tanita Scale SECA 874) to the nearest 0.1 cm and 0.1 kg, respectively. Participants were dressed in minimal, snug clothes without shoes. BMI was calculated by dividing weight in kilograms by the height in square meters. WC was measured at the narrowest part of the abdomen and hip circumference (HC) was measured at the maximal width of the hips. Both WC and HC were recorded to the nearest 0.1 cm. Waist-to-hip ratio (WHR) was calculated by dividing WC by HC. Seated resting blood pressure was measured using an Omron digital blood pressure cuff after the subject had been seated with uncrossed legs for five minutes. Blood pressure measurements were taken twice with a two-minute rest interval between each measurement and results were averaged for analysis.

### 2.3. Statistical Analysis

Results are presented as mean values ± standard deviations (SD). Summary statistics were calculated for demographic characteristics. Shapiro–Wilk W goodness-of-fit test indicated lack of normality, mostly right skewness, on all continuous measurements, so nonparametric tests were used. Spearman’s rho correlation coefficient (ρ) was used to determine the relationship between NC and various anthropometric indices by sex. To find the optimal, maximal summation of sensitivity and specificity for NC, the receiving operating characteristic (ROC) curve analysis was done to determine cut-off points at intervals of 0.5 cm against high levels of WC for males and females. A high WC defined as >102 cm for males and >88 cm for females was described previously [20]. Based on this classification, true-positive subjects were those with high WC and high NC. True-negative subjects were those with low WC and low NC. False-positive subjects were those with low WC and high NC. False negatives were those with high WC and low NC. Sensitivity was calculated as true positives/(true positives + false negatives). Specificity was calculated as true negatives/(true negatives + false positives). *p* < 0.05 was considered statistically significant, however, Benjamini–Hochberg adjustment with false discovery rate 0.25 was utilized for multiple testing. All analyses were conducted using JMP (JMP^®^, Version Pro 12, SAS Institute Inc. Cary, NC, USA, Copyright ©2013).

### 2.4. Ethical Statement

Each university’s IRB approved the study procedures (University of Florida IRB #2014-U-0547 FRUVED; University of Tennessee IRB #9366B; South Dakota State University IRB #1404023EXP; West Virginia University IRB #1409447372; Syracuse University IRB #14-175; Kansas State University IRB #7257; University of Maine IRB #2014-06-21; Auburn University IRB #15-164-EP1504). All participants provided written consent to participate by signing an IRB-approved informed consent form.

## 3. Results

Of the 1562 subjects, 48 were eliminated for missing data. Of the remaining 1514, 1064 (70%) were female and 450 (30%) were male. There was a spread of those from the different regions of the United States where a majority were from the Southeast (43%), followed by Northeast (29%), Midwest (18%), Southwest (3%), and Northwest (1%). About 25% of the students that were assessed came from the University of Florida, followed by University of Tennessee (14.5%), with West Virginia, Maine, and Syracuse each having 12%. Additionally, 82% identified as being from the Non-Appalachian region. In examining the ethnicity spread, 68% were White, 11% were Asian, 11% were Black, and 10% were ‘Other’. The mean age was 19.7 ± 1.4 years. Table 1 shows the anthropometric measures in males and females. The mean BMI was 24.7 ± 4.3 kg/m^2^ in males and 24.2 ± 4.9 kg/m^2^ in females.

Table 2 lists correlations among NC and various anthropometric measures. In both sexes, NC was positively correlated with weight, height, BMI, WC, HC, WHR, and systolic blood pressure (all *p* < 0.0001). In females, NC was also positively correlated with diastolic blood pressure (*p* < 0.0001).

**Figure 1:**
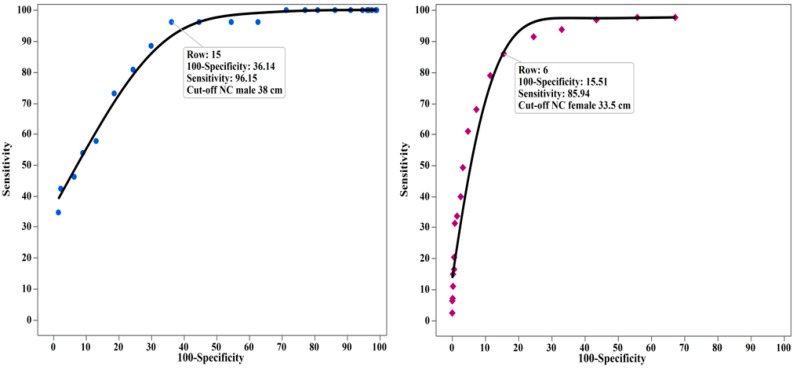
Receiving operating characteristics (ROC) curves determined from the neck circumference and central obesity (waist circumference >102 cm in males (**left**) and >88 cm females (**right**)).

Using ROC analysis (Table 3), an NC ≥ 38 cm in males (sensitivity 0.96, specificity 0.64) and an NC ≥ 33.5 cm in females (sensitivity 0.86, specificity 0.85) were the optimal cut-off levels for determining subjects with central obesity (WC measurement > 102 cm in males and WC > 88 cm in females) (Figure 1).

## 4. Discussion

The current study suggests that NC is a potential indicator for measuring central obesity in young adults in the U.S. NC is well established, but its evaluation is limited in young generations where preventative measures are vital for health maintenance. BMI and WC have been used traditionally as indices for general and visceral obesity, respectively [21,22], and are useful first steps in determining risk of disease with levels and distribution of fat [23]. However, the cut-off values for determining optimal health vary with age, sex, and ethnicity [8]. Additionally, both assessments have their own limitations, where BMI does not account for body fat distribution and cannot distinguish between lean and fat mass. WC requires that subjects be minimally dressed, not eat before the measurement, as well as have an empty bladder. Further, WC measurements are more invasive for patients compared with NC. Some patients have expressed concerns about removing clothing during waist circumference measurements and health care practitioners have reported feeling uncomfortable performing waist circumference measurements perceiving that their patients might feel embarrassed [24].

Results of this study show a strong positive correlation of NC with BMI, WC, and WHR in both female and male subjects. These findings were supported by several studies that examined the association of NC with conventional measurements of obesity [17,25]. In addition, researchers have investigated the same correlations among individuals ages 18 and older. Researchers who studied 41 male and 109 female students (ages 18–20) found a strong positive correlation with BMI, WC, and HC [16]. The only exception was the WHR, which was applicable to males only. Similar results in an older population were found in another study of 979 subjects (460 males and 519 females, ages 30–70) where NC was significantly associated with BMI, age, weight, WC, HC, and WHR [19]. 

An NC ≥ 38 cm for males and ≥33.5 cm in females were identified as cut-off points for those with central obesity (WC > 102 cm in males and >88 cm in females) in our study participants. These cut-offs are higher than those found in studies that used BMI as the reference point. For older adolescents, Hingorgjo et al. found that an NC ≥ 35.5 cm in males and ≥32 cm in females should be considered the cut-off point for overweight/obesity [16]. However, this data used BMI classifications specific to individuals from Asia-Pacific (BMI ≥ 23 kg/m^2^ and BMI ≥ 25 kg/m^2^, for overweight and obese, respectively) so these findings are not applicable to young adults in the U.S. Others have found a cut-off < 37 cm and <34 cm in males and females, respectively [19]. A study conducted on adults (35–65 years), showed, similarly to our study, an NC ≥ 38 cm for males and ≥34 cm for females were optimal cut-off points to determine metabolic syndrome. Comparable to our study of using WC as the reference point, researchers found cut-off points to be ≥38 cm for males and ≥35 cm for females in 3182 adults (ages 20–80) with Type II diabetes [17]. However, this research was conducted in Chinese subjects who have different standards of obesity compared to individuals in the U.S. Thus, the varied cut-off levels found in studies suggest the need to develop standard values for local populations, various disease states, and different ethnicities.

## 5. Conclusions

In summary, our results agree with recent literature and reinforce the novelty of using NC as a predictor of obesity—more specifically, central obesity, which is closely associated with metabolic disease. Unlike other anthropometric measures, such as WC, NC is simple, noninvasive, and inexpensive, and can also be used with larger sample sizes. Although NC shows a strong correlation with other measures of obesity, it should be considered as a screening test for early interdisciplinary prevention in young adults. Our study suggests that young adult males with an NC < 38 cm and females with an NC < 33.5 cm do not require additional evaluation and individuals above these levels should seek further evaluation of adiposity and body distribution.

A few limitations are present in the current study. Although we had a large sample size with some spread of different ethnic backgrounds and individuals across the U.S., there was still a small sample of males. Secondly, our convenience sample consisted of mostly young adults who were healthy, therefore the results cannot be generalizable to other populations with more diversity and higher prevalence of disease and ethnicity. Additionally, this was a cross-sectional study with pre-existing measures, which makes it difficult to determine a causal reference. Therefore, a longitudinal study may be further warranted in the young adult population. Despite these limitations, we were able to make assumptions regarding the usefulness of NC in diagnosing central obesity and metabolic disorders in young adults. Future research would benefit from using other samples of college-age students across the nation with more metabolic abnormalities. However, our research results can be used as a starting point for comprehensive testing in young adults with increased risk of disease.

## Figures and Tables

**Table 1:** Subject characteristics.

Variables	Males	Females
Age (years)	19.6 ± 1.4 (18–27)	19.8 ± 1.4 (18–28)
Weight (kg)	76.2 ± 15.7 (42.5–153.6)	65.7 ± 15.3 (39.3–146.2)
Height (cm)	175.3 ± 7.7 (146.5–197.5)	164.6 ± 7.1 (141.2–191.2)
BMI (kg/m^2^)	24.7 ± 4.3 (14.7–41.8)	24.2 ± 4.9 (16.3–49.7)
Waist Circumference (cm)	83.3 ± 11.1 (61.5–139.5)	76.8 ± 11.2 (56.0–156.3)
Hip Circumference (cm)	101.1 ± 9.7 (69.4–144.0)	99.8 ± 10.4 (63.4–149.0)
Neck Circumference (cm)	37.4 ± 2.7 (29.4–48.5)	32.3 ± 2.4 (26.8–50.0)
Systolic Blood Pressure (mm/Hg)	118 ± 13 (74–162)	106 ± 11 (69–166)
Diastolic Blood Pressure (mm/Hg)	68 ± 9 (46–96)	68 ± 9 (43–107)

Values are means ± SD (Range).

**Table 2:** Relationships between neck circumference and various anthropometric variables.

Variables	Males		Females	
	ρ	*p*-Value	ρ	*p*-Value
Age (years)	0.04	0.45	−0.06	0.07 *
Weight (kg)	0.69	<0.0001 **	0.71	<0.0001 **
Height (cm)	0.26	<0.0001 **	0.28	<0.0001 **
BMI (kg/m^2^)	0.70	<0.0001 **	0.69	<0.0001 **
Waist Circumference (cm)	0.69	<0.0001 **	0.72	<0.0001 **
Hip Circumference (cm)	0.64	<0.0001 **	0.69	<0.0001 **
Waist-to-Hip Ratio	0.46	<0.0001 **	0.41	<0.0001 **
Systolic Blood Pressure (mm/Hg)	0.25	<0.0001 **	0.31	<0.0001 **
Diastolic Blood Pressure (mm/Hg)	0.04	0.42	0.22	<0.0001 **

** Correlation is significant at 0.05; * Correlation is significant at False Discovery Rate of 0.25 after Benjamini–Hochberg adjustment to multiple analyses.

**Table 3:** Neck circumference (cm) cut-off levels for determining the subjects with waist circumference >102 cm in males and >88 cm females.

	Males		Females	
Cut-Off (cm)	Sensitivity (%)	Specificity (%)	Sensitivity (%)	Specificity (%)
31	100	1.0	97.7	32.8
31.5	100	1.2	97.7	44.2
32	100	2.4	96.9	56.6
32.5	100	3.4	93.8	67.0
33	100	3.9	91.4	75.5
33.5	100	5.3	**85.9**	**84.5**
34	100	8.9	78.9	88.6
34.5	100	13.7	68.0	92.7
35	100	19.0	60.9	95.3
35.5	100	22.9	49.2	96.8
36	100	28.7	39.8	97.5
36.5	96.2	37.4	33.6	98.6
37	96.2	45.5	31.3	99.2
37.5	96.2	55.4	20.3	99.5
38	**96.2**	**63.9**	16.4	99.5
38.5	88.5	70.1	14.8	99.8
39	80.8	75.7	10.9	99.8
39.5	73.1	81.5	7.0	99.8
40	57.7	87.0	6.3	100
40.5	53.9	91.1	2.3	100
41	46.2	93.7	2.3	100
41.5	42.3	97.8	2.3	100
42	34.6	98.6	2.3	100

Bold values indicate the optimal cut-off NC levels based on maximum of (Sensitivity + Specificity) for determining subjects with central obesity (WC measurement > 102 cm, NC ≥ 38 cm in males and WC > 88 cm, NC ≥ 33.5 cm in females).

## Availability of Data and Materials

The datasets used and analyzed during the current study are available from the corresponding author on reasonable request.

## Abbreviations

NC	Neck circumference
BMI	Body mass index
WC	waist circumference
HC	hip circumference
SBP	systolic blood pressure
U.S.	United States
HDL	high-density lipoprotein
WHR	waist-to-hip ratio
ROC	receiving operating characteristic curve
